# Biodata Mining of Differentially Expressed Genes between Acute Myocardial Infarction and Unstable Angina Based on Integrated Bioinformatics

**DOI:** 10.1155/2021/5584681

**Published:** 2021-09-13

**Authors:** Siyu Guo, Zhihong Huang, Xinkui Liu, Jingyuan Zhang, Peizhi Ye, Chao Wu, Shan Lu, Shanshan Jia, Xiaomeng Zhang, Xiuping Chen, Miaomiao Wang, Jiarui Wu

**Affiliations:** ^1^Beijing University of Chinese Medicine, Beijing 100102, China; ^2^National Cancer Center, National Clinical Research Center for Cancer, Chinese Medicine Department of the Cancer Hospital of the Chinese Academy of Medical Sciences and Peking Union Medical College, Beijing 100021, China; ^3^Institute of Chinese Medical Sciences, State Key Laboratory of Quality Research in Chinese Medicine, University of Macau, Macau, China

## Abstract

Acute coronary syndrome (ACS) is a complex syndrome of clinical symptoms. In order to accurately diagnose the type of disease in ACS patients, this study is aimed at exploring the differentially expressed genes (DEGs) and biological pathways between acute myocardial infarction (AMI) and unstable angina (UA). The GSE29111 and GSE60993 datasets containing microarray data from AMI and UA patients were downloaded from the Gene Expression Omnibus (GEO) database. DEG analysis of these 2 datasets is performed using the “limma” package in R software. DEGs were also analyzed using protein-protein interaction (PPI), Molecular Complex Detection (MCODE) algorithm, Gene Ontology (GO), and Kyoto Encyclopedia of Genes and Genomes (KEGG) enrichment analysis. Correlation analysis and “cytoHubba” were used to analyze the hub genes. A total of 286 DEGs were obtained from GSE29111 and GSE60993, including 132 upregulated genes and 154 downregulated genes. Subsequent comprehensive analysis identified 20 key genes that may be related to the occurrence and development of AMI and UA and were involved in the inflammatory response, interaction of neuroactive ligand-receptor, calcium signaling pathway, inflammatory mediator regulation of TRP channels, viral protein interaction with cytokine and cytokine receptor, human cytomegalovirus infection, and cytokine-cytokine receptor interaction pathway. The integrated bioinformatical analysis could improve our understanding of DEGs between AMI and UA. The results of this study might provide a new perspective and reference for the early diagnosis and treatment of ACS.

## 1. Introduction

Acute coronary syndrome (ACS) is a complex syndrome of clinical symptoms, which is characterized by acute myocardial ischemia with severe coronary stenosis or occlusion caused by coronary plaque rupture and subsequent thrombosis, mainly including acute myocardial infarction (AMI) and unstable angina (UA) [1, 2]. AMI can be divided into acute ST-segment elevation acute myocardial infarction (STEMI) and acute non-ST-segment elevation acute coronary syndrome (NSTEMI) due to the different stratification of early diagnosis and treatment [3]. According to the statistics of the American Heart Association (AHA) and the National Institutes of Health in 2019, there were about 633,000 patients with acute coronary syndrome in 2014, including about 609,000 AMI patients and about 24,000 UA patients [4]. In addition, a study showed that the incidence of AMI had been increasing in younger populations and decreasing in the elderly and that improvement in the in-hospital mortality of AMI may have reached a plateau in all age groups in Japan [5].

Based on clinical epidemiology, the mechanism of the relationship between UA and AMI was studied, and great progress has been made [6–9]. Studies have shown that some UA patients will develop into AMI if they are not treated properly [10, 11]. Therefore, the identification of differential genes (DEGs) between UA and AMI is essential for the development of new targeted drugs to improve clinical efficacy.

**Figure 1:**
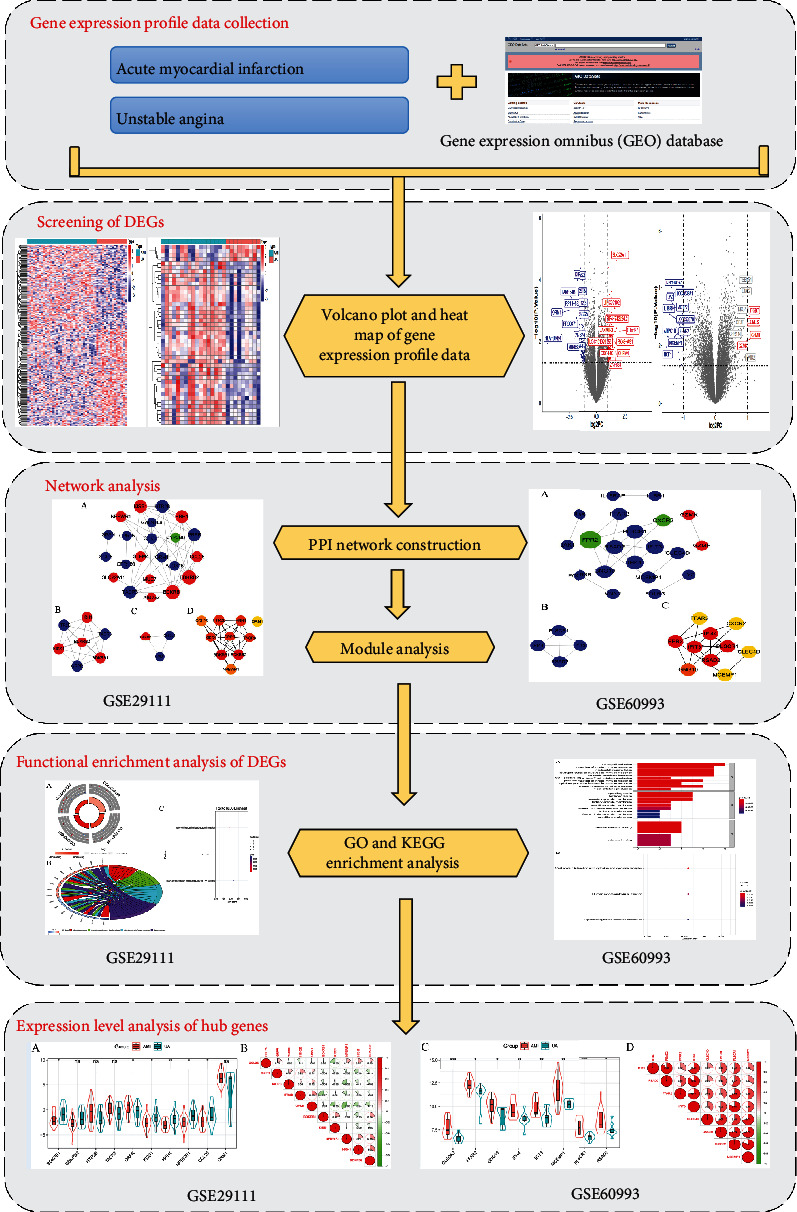
Integrated bioinformatics-based technical route for differential gene research between acute myocardial infarction and unstable angina pectoris.

With the development of high-throughput gene chip and transcriptome sequencing methods, more and more gene chip technologies are used in the research of cancer and cardiovascular diseases, which enriches the biological markers for early diagnosis, classification, and prognosis of diseases and also provides references for the targeted therapy of diseases [12–15]. Microarray technology is conducive to understanding gene association, location, expression, and linkage studies and is widely used to compare DEGs in patients with different diseases [16]. At present, although there have been some bioinformatics-based studies on the miRNA, DEGs, and mRNA of AMI, the role of DEGs between AMI and UA remains unclear [17, 18]. In this study, the microarray data containing both AMI and UA were downloaded from the GEO database, and the integrated bioinformatics method was adopted to screen the DEGs, protein-protein interaction (PPI), and related key pathways between AMI and UA. This research may provide new insights for understanding the functional regulation mechanism of DEGs between AMI and UA and help to develop relevant targeted therapies in further in-depth work. The flow chart of integrated bioinformatics research is shown in Figure 1.

## 2. Materials and Methods

### 2.1. Gene Expression Profile Data Collection

The GEO database (https://www.ncbi.nlm.nih.gov/gds/) developed and maintained by the National Center for Biotechnology Information (NCBI) not only collects and shares publicly available transcriptome data but also facilitates biomedical research [19]. In the current study, “Acute myocardial infarction” and “Unstable angina” were used as keywords to obtain gene expression microarray datasets related to AMI and UA. The criteria of the chip included are as follows: (1) the sample source of the dataset is human, (2) the dataset includes AMI and UA samples, and (3) there are no less than 20 samples per dataset.

### 2.2. Screening of DEGs

First, the gene expression dataset is translated into common gene symbols by using annotation information from the platform. The “limma” package of R software was utilized to screen the original dataset for expression normalization and differential expression analysis [20]. In addition, the actual situation determines whether the normalized data is log2 converted. The heatmap and volcano map show data results. The threshold of DEG screening was ∣log2 FC | ≥1 and *P* < 0.05 (FC: fold change).

### 2.3. Protein Interaction Analysis and Module Analysis of DEGs

The interaction between proteins is crucial for understanding the metabolic and molecular mechanisms of diseases. The number of organisms covered by the STRING database (http://string-db.org, version: 11.0) is more than double to 5090 compared with the previous version. It collects, scores, and integrates all public sources of protein-protein interaction information and supplements this information through computational predictions, including direct and indirect correlations between proteins [21]. In this study, DEGs were input into STRING, and the species was set as “Homo sapiens.” In addition, the data with a confidence score greater than 0.7 was selected (low confidence: score > 0.15, moderate confidence: score > 0.4, and high confidence: score > 0.7). The results were imported into Cytoscape 3.7.1 (https://cytoscape.org/) to establish a protein-protein interaction (PPI) network model [22], and the MCODE (Molecular Complex Detection) plug-in was used for module analysis [23]. The parameters were set to the default values (degree cutoff = 2, node score cutoff = 0.2, *K*‐core = 2, and Max.depth = 100). Then, the Maximal Clique Centrality (MCC) algorithm in “cytoHubba” was applied to screen the hub genes with high connectivity in PPI networks [24].

### 2.4. Enrichment Analysis of DEGs

GO provides a framework for describing the function of gene products in organisms, which can identify the biological characteristics of high-throughput genome and transcriptome data, including biological process (BP), cellular component (CC), and molecular function (MF) [25]. KEGG can be used in the functional interpretation and practical application of DEG information [26]. In order to reveal the biological significance of DEGs, DAVID v6.8 (https://david.ncifcrf.gov/), “clusterProfiler” [27], and “GOplot” packages [28] in R software were utilized for GO function and KEGG pathway enrichment analysis, and the results with FDR < 0.05 were selected for analysis and visualization.

### 2.5. Expression Level Analysis and Correlation Analysis of Key DEGs

According to the MCC algorithm, 10 genes with the most stable and highest score in the network were selected as the key genes. The differences in hub gene expression between AMI and UA were shown using the boxplot tool in ImageGP (http://www.ehbio.com/ImageGP/). In addition, in order to better understand the key DEGs, the Pearson correlation coefficient was used for correlation analysis, and the “Corrplot” package in R software was used for visualization [29, 30].

## 3. Results

### 3.1. Screening of Differentially Expressed Genes

**Figure 2:**
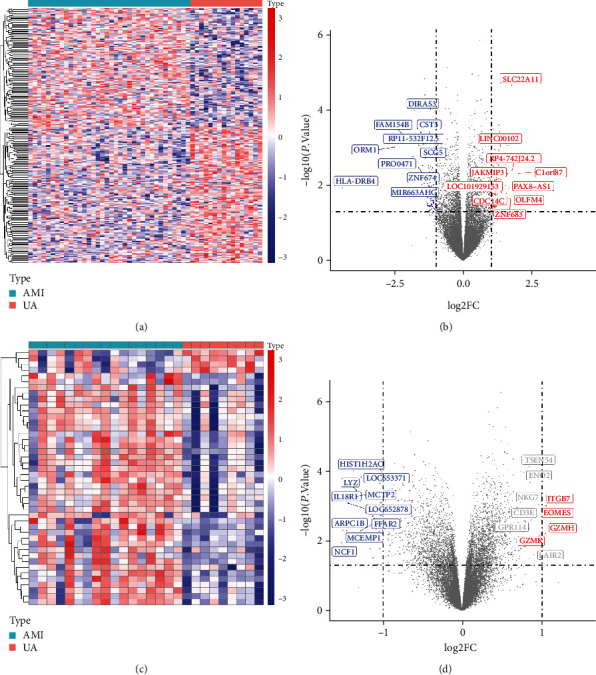
Heatmaps and volcano plots of differentially expressed genes between GSE29111 and GSE60993. (a) Heatmap of upregulated and downregulated genes of GSE29111. Each column represents a dataset, and each row represents a gene. Blue represents the downregulated gene, and red represents the upregulated gene. (b) Volcano plot of GSE29111. Blue represents the downregulated gene, red represents the upregulated gene, and gray represents the remaining differentially expressed genes. (c) Heatmap of upregulated and downregulated genes of GSE60993. (d) Volcano plot of GSE60993.

The present study finally included 2 datasets (GSE29111 and GSE60993), each of which contained AMI and UA samples, including 53 AMI samples and 25 UA samples (Table 1). DEG analysis of these 2 datasets is performed using the “limma” package in R software. A total of 242 differential genes were obtained from GSE29111, including 128 upregulated genes and 114 downregulated genes (Supplementary Table 1). GSE60993 yielded 44 differentially expressed genes, including 4 upregulated genes and 40 downregulated genes (Supplementary Table 2).  Figure 2 shows the DEG volcano plots and heatmaps of the included dataset.

### 3.2. Protein Interaction Analysis and Module Analysis of DEGs

**Figure 3:**
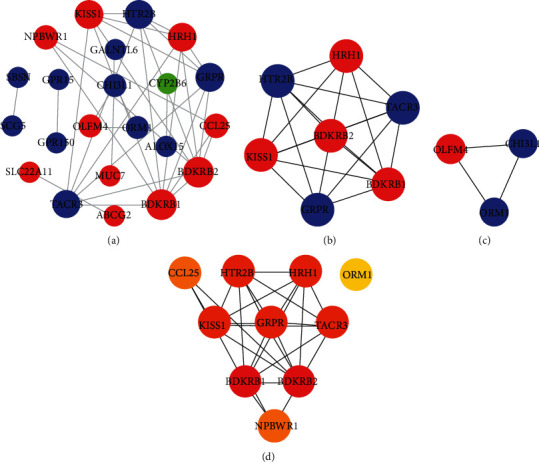
Protein-protein interaction (PPI) network and hub clustering modules of GSE29111. (a) The PPI network of DEGs. (b) Module 1 (MCODE score = 7). (c) Module 2 (MCODE score = 3). Blue represents the downregulated gene, and red represents the upregulated gene. The size of the node is proportional to the degree. (d) The connectivity map of 10 hub genes. From yellow to red, the darker the color, the higher the MCC score.

The obtained DEGs were imported into the STRING database, and the data with a confidence score greater than 0.7 were selected. Cytoscape was used for network visualization analysis of these interacting DEGs. The PPI network of GSE29111 includes 22 nodes and 34 edges (Figure 3(a)). Using MCODE plug-in module analysis, a total of 2 significant modules were obtained (MCODE score > 3) (Figures 3(b) and 3(c)). Module 1 contains 7 genes (4 upregulated and 3 downregulated), and module 2 contains 3 genes (1 upregulated and 2 downregulated). In addition, the PPI network is analyzed by the plug-in “cytoHubba” of Cytoscape. According to the MCC algorithm, 10 genes with the most stable and highest score in the network were selected as the hub genes: B2 bradykinin receptor (BDKRB2), histamine H1 receptor (HRH1), metastasis-suppressor KiSS-1 (KISS1), gastrin-releasing peptide receptor (GRPR), neuromedin-K receptor (TACR3), 5-hydroxytryptamine receptor 2B (HTR2B), C-C motif chemokine 25 (CCL25), neuropeptide B/W receptor type 1 (NPBWR1), B1 bradykinin receptor (BDKRB1), and alpha-1-acid glycoprotein 1 (ORM1) (Table 2 and Figure 3(d)).

**Figure 4:**
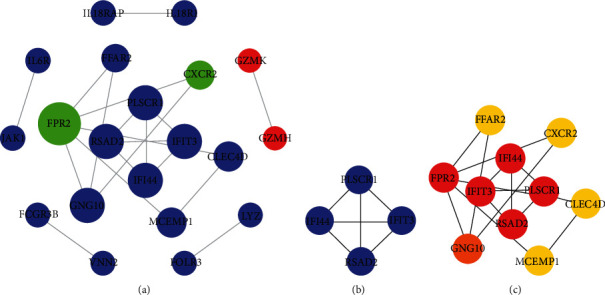
Protein-protein interaction (PPI) network and hub clustering modules of GSE60993. (a) The PPI network of DEGs. (b) Module 1 (MCODE score = 4). Blue represents the downregulated gene, and red represents the upregulated gene. The size of the node is proportional to the degree. (c) The connectivity map of 10 core genes. From yellow to red, the darker the color, the higher the MCC score.

The PPI network of GSE60993 includes 20 nodes and 19 edges (Figure 4(a)). The MCODE plug-in was used for module analysis, and a significant module (score = 4) was obtained. The genes included were downregulated genes (Figure 4(b)). The PPI network was analyzed by Cytoscape plug-in “cytoHubba.” Based on the MCC algorithm, the 10 most stable and scored genes in the network were selected as the core genes: interferon-induced protein 44 (IFI44), free fatty acid receptor 2 (FFAR2), guanine nucleotide-binding protein G(I)/G(S)/G(O) subunit gamma-10 (GNG10), C-X-C chemokine receptor type 2 (CXCR2), mast cell-expressed membrane protein 1 (MCEMP1), N-formyl peptide receptor 2 (FPR2), C-type lectin domain family 4 member D (CLEC4D), interferon-induced protein with tetratricopeptide repeats 3 (IFIT3), radical S-adenosyl methionine domain-containing protein 2 (RSAD2), and phospholipid scramblase 1 (PLSCR1) (Table 3 and Figure 4(c)).

### 3.3. Enrichment Analysis of DEGs

**Figure 5:**
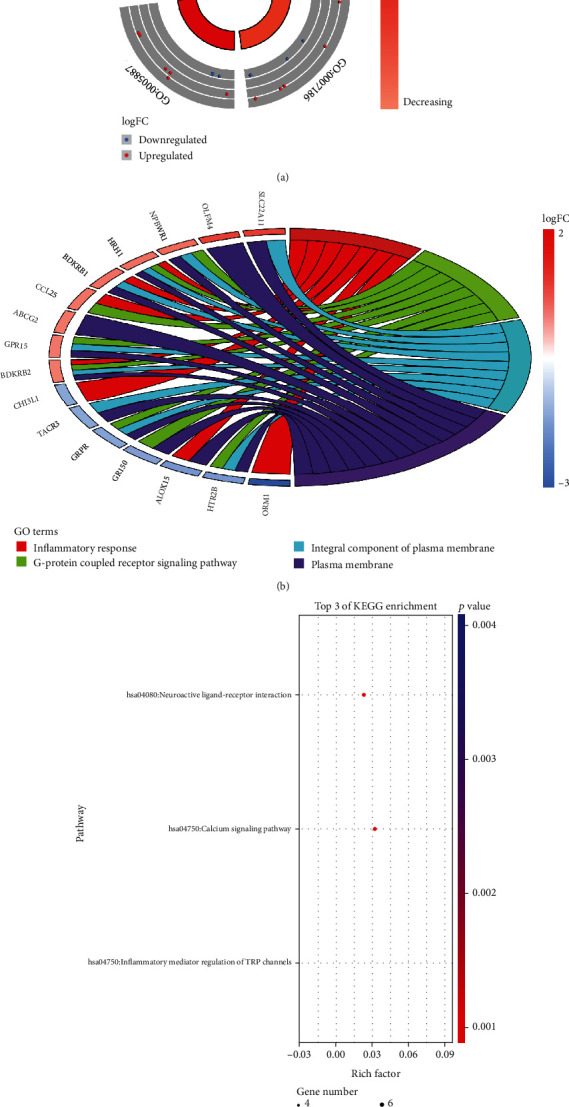
GO and KEGG pathway enrichment analysis of DEGs in GSE29111. (a) Circular plot for GO enrichment analysis of DEGs. The inner circle represents the *z*-score, which is the downregulation of enriched genes under a certain GO entry. The outer circle represents GO item ID. The points in the outer ring represent the distribution of downregulated genes involved in this item. (b) Chord plot for GO enrichment analysis of DEGs. The right side represents GO entries, and different colors represent different GO entries. The left side represents genes enriched in GO entries. (c) KEGG pathway enrichment analysis of DEGs. The horizontal axis represents the rich factor, and the vertical axis represents the KEGG pathway.

GO and KEGG pathway enrichment analysis was performed on PPI data of GSE29111 differentially expressed genes using DAVID and “GOplot” package in R software. According to FDR < 0.05, 4 GO entries with significant enrichment were selected (Figures 5(a) and 5(b), Table 4). It contains 2 BP items and 2 CC items: inflammatory response, G-protein-coupled receptor signaling pathway, integral component of plasma membrane, and plasma membrane. According to FDR < 0.05, 3 significantly enriched KEGG pathways were screened: neuroactive ligand-receptor interaction, calcium signaling pathway, and inflammatory mediator regulation of TRP channels (Figure 5(c)).

**Figure 6:**
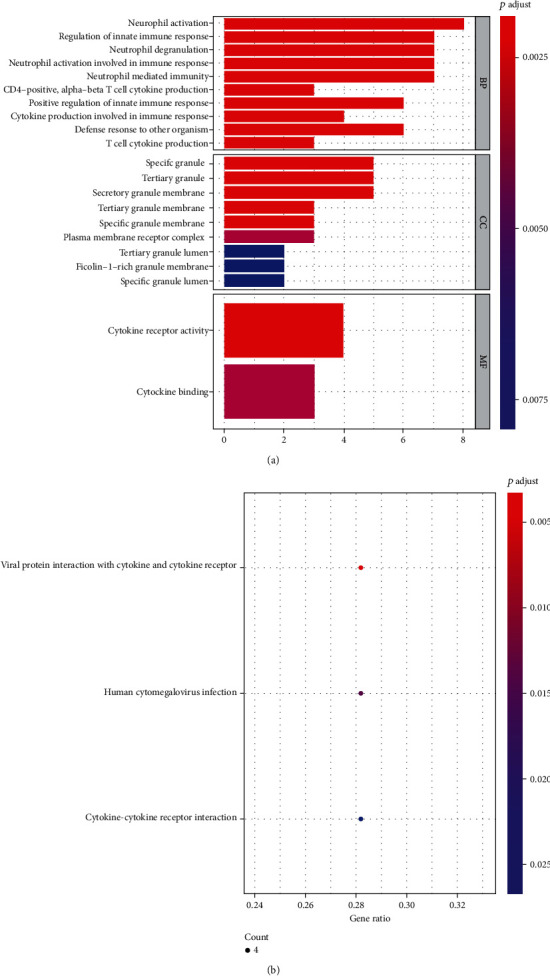
GO and KEGG pathway enrichment analysis of DEGs in GSE60993. (a) GO enrichment analysis of DEGs. (b) KEGG pathway enrichment analysis of DEGs. The horizontal axis represents gene ratio, which is the ratio of the number of genes associated with the pathway to the total number of DEGs. The vertical axis represents the KEGG pathway.

In the current study, GO and KEGG pathway enrichment analysis was performed on PPI data of GSE60993 differentially expressed genes through the “clusterProfiler” package. According to *P* adjust < 0.01, a total of 52 significantly enriched GO entries were screened (Figure 6(a) and Table 5). There are 41 BP items, mainly related to neutrophil activation, immune response, and inflammatory response; 9 CC items, primarily relevant to particles and plasma membrane; and 2 MF items, basically associated with cytokine receptor activity and cytokine binding. According to *P* adjust < 0.05, 3 significantly enriched KEGG pathways were screened: viral protein interaction with cytokine and cytokine receptor, human cytomegalovirus infection, and cytokine-cytokine receptor interaction (Figure 6(b)).

### 3.4. Expression Level and Correlation Analysis of Key DEGs

**Figure 7:**
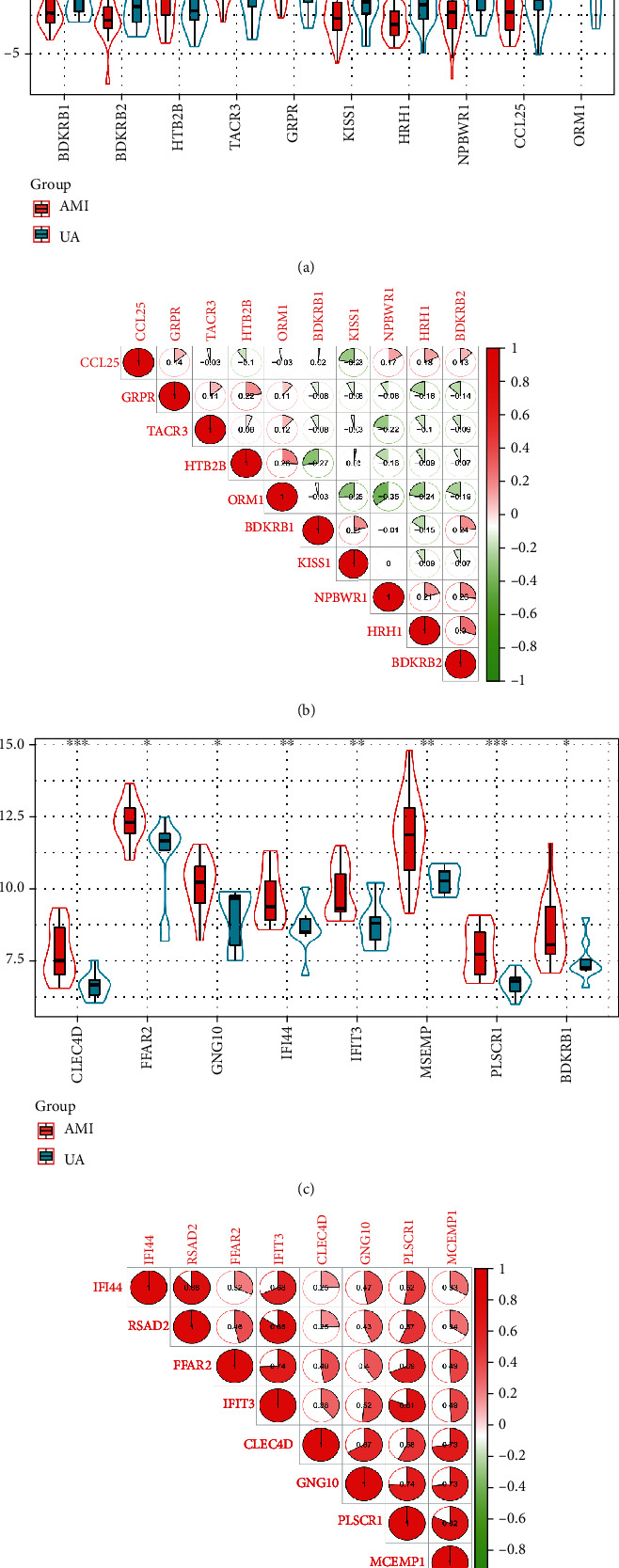
Expression level analysis of key genes in GSE29111 and GSE60993. (a) Expression analysis of GSE29111 key genes in the AMI and UA groups. (b) Bitmap for correlation analysis of key genes in GSE29111. (c) Expression analysis of GSE60993 key genes in the AMI and UA groups. (d) Bitmap for correlation analysis of key genes in GSE60993. ns: there is no statistically significant difference between groups. ^∗^*P* < 0.05; ^∗∗^*P* < 0.01; ^∗∗∗^*P* < 0.001.

In order to better understand the key DEGs screened by GSE29111 and GSE60993, their expression levels and correlation analysis were performed. According to Pearson's correlation coefficient (*r*) classification, the absolute values of 0-0.30, 0.30-0.50, 0.50-0.70, and 0.70-1.00 indicated “poor,” “medium,” “good,” and “strong” correlations. In addition, “*r* = 0” means completely irrelevant and “*r* = 1.00” means completely relevant. The key DEGs screened by GSE29111, BDKRB1, BDKRB2, CCL25, HRH1, KISS1, and NPBWR1 were highly expressed in UA patients and lowly expressed in AMI patients. GRPR, TACR3, HTR2B, and ORM1 were lowly expressed in UA patients and highly expressed in AMI patients (Figure 7(a)). As shown in Figure 7(b), the correlations between BDKRB1, BDKRB2, CCL25, HRH1, KISS1, NPBWR1, GRPR, TACR3, HTR2B, and ORM1 were poor or moderate.

The key DEGs screened by GSE60993, CLEC4D, FFAR2, GNG10, IFI44, IFIT3, MCEMP1, PLSCR1, and RSAD2 were highly expressed in UA patients and lowly expressed in AMI patients (Figure 7(c)). As shown in Figure 7(d), CLEC4D, FFAR2, GNG10, IFI44, IFIT3, MCEMP1, PLSCR1, and RSAD2 were positively correlated. Among them, PLSCR1 showed a strong positive correlation with MCEMP1 (*r* = 0.82). IFI44, RSAD2, and CLEC4D showed a poor positive correlation (*r* = 0.25).

## 4. Discussion

Many factors affect the occurrence and development of ACS, such as gender, fatigue, and frailty [31–33]. According to “China cardiovascular disease report 2018,” the mortality rate of AMI showed an overall upward trend from 2002 to 2016, and the mortality rate in rural areas was higher than that in urban areas many times. Although a lot of research has been conducted on the molecular mechanisms related to AMI and UA, the understanding of the differentially expressed genes between AMI and UA is insufficient. Therefore, it is still a challenge to diagnose the disease types of ACS patients quickly and accurately. Microarray analysis is a powerful tool for screening hub genes, which can effectively reduce candidate genes associated with multifactorial diseases and identify potential molecular mechanisms of disease and biomarkers for diagnosis and prognosis [34, 35]. In this study, we obtained the microarray datasets of AMI and UA patients from the GEO database to clarify their DEGs and candidate biomarker.

The integrated bioinformatics analysis method was performed to screen the potential differential genes related to AMI and UA. Through retrieval, 2 datasets (GSE29111 and GSE60993) from the GEO database were included. Although datasets as GSE29111 and GSE60993 were already used and published by some authors, the main comparison is the DEGs between AMI and healthy controls. The difference is that this study used these two datasets to explore the DEGs between AMI and UA. A total of 242 DEGs were obtained from GSE29111, including 128 upregulated genes and 114 downregulated genes. There were 44 DEGs obtained from GSE60993, including 4 upregulated genes and 40 downregulated genes. According to the MCC algorithm, the 10 most stable and highest score genes in the PPI network were selected as core genes. The hub genes involved in GSE29111 included BDKRB1, BDKRB2, CCL25, HRH1, KISS1, NPBWR1, GRPR, TACR3, HTR2B, and ORM1. The core genes involved in GSE60993 include IFI44, FFAR2, GNG10, CXCR2, MCEMP1, FPR2, CLEC4D, IFIT3, RSAD2, and PLSCR1. These identified core genes act as a whole and may play an important role in the early diagnosis of ACS.

GO enrichment analysis showed that the DEGs screened from GSE29111 were mainly enriched in the inflammatory response, G-protein-coupled receptor signaling pathway, integral component of plasma membrane, and plasma membrane. The GO entries of the DEGs screened from the GSE60993 were mainly related to neutrophil activation, immune response, inflammatory response, particles, plasma membrane, cytokine receptor activity, and cytokine binding. Over the past decades, the critical role of immune inflammatory processes in the occurrence, development, and deterioration of many cardiovascular diseases has received increasing attention, such as atherosclerosis, myocardial infarction, heart failure, myocarditis, and vasculitis [36, 37]. At present, many biological, immunoregulatory, and antioxidant strategies have been proposed for heart protection [38]. Ong et al. summarized the inflammatory response after AMI; the results showed that the attack of acute myocardial ischemia can cause cell damage and death of different myocardial components [39]. Moreover, it triggers acute proinflammatory responses through a synergistic effect of multiple processes, which leads to the release of a variety of proinflammatory mediators and induces the recruitment of inflammatory cells to the infarction area [40]. In the animal model of AMI, the injured myocardial cells released interleukin-1 alpha (IL-1*α*), while interleukin-1 beta (IL-1*β*) increased after infarction [41]. The findings of our study suggest that the DEGs between AMI and UA are related to the inflammatory response, which provides a reference for their treatment.

The enrichment analysis of the KEGG pathway showed that the main enriched pathways of DEGs screened from the GSE29111 dataset were related to the interaction of neuroactive ligand-receptor (hsa04080), calcium signaling pathway (hsa04020), and inflammatory mediator regulation of TRP channels (hsa04750). Previous studies have shown that the nervous system regulation of cardiac activity has nothing to do with the physiological state of the heart [42]. However, the autonomic balance between sympathetic and parasympathetic systems plays an important role in the regulation of the cardiovascular system [43]. The facts show that the interruption of cardiac parasympathetic (vagus) activity is a common feature of various cardiovascular diseases including AMI [44]. In addition, some animal experiments have shown that increased vagus nerve activity has a protective effect on the heart of myocardial ischemia/reperfusion injury [45, 46]. Wang et al. showed that in patients undergoing coronary artery bypass grafting, the cardioprotective effect of sevoflurane was related to the interaction of neuroactive ligand-receptor [47]. Chen et al. showed that the neuroactive ligand-receptor interaction pathway was closely related to human arrhythmogenic right ventricular cardiomyopathy [48]. Although the correlation between AMI and neuroactive ligand-receptor interaction has not been reported, this pathway may be a new mechanism of AMI, which is worth further study.

Calcium, as a common intracellular second messenger, is involved in regulating fertilization, gene transcription, secretion, and myocardial cells [49]. Ruiz-Meana et al. showed that calcium increased during myocardial infarction [50]. Garcia-Dorado et al. proposed to develop effective and reliable treatments to inhibit Ca^2+^-mediated myocardial cell death in patients with myocardial infarction, which has important therapeutic significance and potential clinical effects [51]. Consistent with our results, the calcium signaling pathway was found to be one of the most significant pathways. Recent studies have shown that the regulation of TRP channels mainly occurs at transcription, translation, and posttranslation levels and depends on ion balance, microbial ligands, cytokines, or reactive oxygen species (ROS). In fact, inflammatory transcription factors such as nuclear factor-*κ*B (NF-*κ*B), signal transduction and transcription activation factor 3 (STAT3), and hypoxia-inducible factor-1 (NIF-1) are all related to the increase of ROS and intracellular Ca^2+^ concentration, and these characteristics make TRP channels effectively regulate inflammation [52]. Although neuroactive ligand-receptor interaction, calcium signaling pathway, and inflammatory mediator regulation of TRP channels have little research on ACS, they may be new ways of occurrence and development of AMI and UA, which is worthy of further study.

In the current study, the core genes enriched in the neuroactive ligand-receptor interaction with the smallest FDR value were discussed and analyzed in detail. HRH1, NPBWR1, BDKRB2, HTR2B, TACR3, BDKRB1, and GRPR are 7 core genes enriched in the neuroactive ligand-receptor interaction. Histamine receptor H1 (HRH1) belongs to the G-protein-coupled receptor family. Its gene products are expressed in many tissues such as smooth muscle and neurons [53, 54]. Bhuiyan et al. found that HRH1 is mainly expressed in the neurons of the solitary tract nucleus, and histamine in the solitary tract nucleus may play a role in regulating cardiovascular homeostasis by activating H1 receptors expressed in the neurons of the solitary tract nucleus [55]. One study showed that the activation of HRH1 drove the formation of atherosclerotic lesions by increasing the vascular permeability of low-density lipoprotein [56]. Neuropeptide W (NPW) and neuropeptide B (NPB) are 2 structural and functional regulatory peptides. They are highly expressed in some brain regions and peripheral tissues (such as the heart, kidney, and stomach), but their distribution in tissues is not similar [57, 58]. In addition, they act on target tissues through neuropeptide B/W receptor 1 (NPBWR1) and neuropeptide B/W receptor 2 (NPBWR2). NPB activates NPBWR1, while NPW stimulates the 2 receptors with similar potency and has different binding affinities [59, 60]. NPW increases the calcium flow into the cells by acting on NPBWR1 and plays a role in regulating vascular myofibrillar tension, so it may be involved in the development of hypertension [61]. In a rat model, the results suggest that central NPW30 can increase sympathetic outflow and affect cardiovascular function [62]. In the past few years, a large number of literatures have shown that B1 bradykinin receptor (BDKRB1) and B2 bradykinin receptor (BDKRB2) are involved in a variety of hemodynamics and metabolism [63]. Marketou et al. found that early use of selective BDKRB2 agonist in the treatment of AMI in mice could reduce tissue damage and improve the therapeutic effect of cardiac remodeling [64]. Therefore, the therapeutic value and potential risk strategy of BDKRB2 agonist in AMI deserve further study.

As a neurotransmitter, 5-hydroxytryptamine (5-HT) not only plays a signal transduction role in immune cells but also regulates mood, behavior, and cardiovascular and gastrointestinal functions [65, 66]. A study showed that 5-HT could regulate macrophage-mediated angiogenesis by reducing the expression of matrix metalloproteinase 12 in tumor-infiltrating macrophages [67]. In addition, it plays a role in the development of pulmonary hypertension depending on the expression of 5-hydroxytryptamine receptor 2B (HTR2B) in bone marrow progenitor cells [68]. de Las et al. showed that 5-HT could regulate macrophage polarization and maintain an anti-inflammatory state through 5-HT2B and 5-HT7 [69]. TACR3 is an important receptor protein, is expressed in the central nervous system, and activated the phosphatidylinositol-calcium second messenger system [70, 71]. In addition, the involvement of TACR3 in human reproductive systems such as normal hypogonadism is evident [72]. It has been reported that neurokinin B (endogenous ligand of TACR3) is involved in the pathogenesis of Parkinson's disease [73]. Schooling's findings suggest that drugs with TACR3 antagonists can regulate the reproductive axis to reduce cardiovascular morbidity and mortality [74]. Additionally, in our study, we observed the TACR3 upregulated in the AMI patients in comparison to UA controls. Gastrin-releasing peptide (GRP) is a neuropeptide whose receptor GRPR is expressed in a variety of cell types and can activate signaling pathways that induce neutrophil migration in vitro and in vivo [75]. In addition, GRPR was found to be expressed in immune cells [76]. Recent studies have shown that selective GRPR antagonist (RC-3095) has anti-inflammatory effects on arthritis and sepsis models [77, 78]. Interestingly, our research identified that HRH1, NPBWR1, BDKRB2, HTR2B, TACR3, BDKRB1, and GRPR are closely related to inflammation. This suggests that anti-inflammatory measures may be needed to treat AMI and UA.

The KEGG pathway enriched by the DEGs screened from the GSE60993 dataset is related to viral protein interaction with cytokine and cytokine receptor (hsa04061), human cytomegalovirus infection (hsa05163), and cytokine-cytokine receptor interaction (hsa04060). Cytokines are extracellular molecules that transmit intercellular signals, mainly involved in cell differentiation and inflammation through binding to specific receptors on the cell surface [79]. Inflammation plays an important role in different stages of coronary atherosclerosis, such as plaque formation, progression, stability, rupture, and acute thrombosis [80]. The combined action of neutrophils and mononuclear macrophages and inflammatory factors promotes the occurrence and development of atherosclerosis and acute coronary events [81]. It has been reported that some functions of mononuclear macrophages and neutrophils are closely related to many cytokines expressed on their surface and corresponding receptors, such as adhesion, chemotaxis, and phagocytosis [82, 83]. Many cytokines related to inflammation are involved in the development of ACS, such as TNF-*α* and IL-6 [84]. Boekholdt et al. showed that the increase of the IL-8 level was associated with the increased risk of coronary artery disease [85]. Therefore, we speculate that the role of the cytokine-cytokine receptor interaction signaling pathway in the progression of AMI and UA may be achieved by regulating the inflammatory response.

In this study, the hub genes enriched in these 3 pathways are C-X-C chemokine receptor type 2 (CXCR2), the receptor of interleukin-8 (IL-8). Neutrophils play an important role in innate immunity [86]. CXC chemokines and related receptors have strong effects on chemotaxis and neutrophil activation, allowing them to migrate to infected or injured sites [87]. Yan et al. detected and analyzed the differential gene expression between AMI patients and stable angina patients and found that the mRNA expression related to CXCR2 in AMI patients was significantly upregulated compared with stable angina patients [88]. A number of studies have shown that the occurrence and development of coronary atherosclerotic heart disease are closely related to monocyte chemoattractant protein 1 (MCP-1), IL-8, and other major chemokines [89–91]. This suggests that the vital DEGs play essential roles in the development and progression of AMI and UA via different signaling pathways. Collectively, most of the DEGs were inflammation-related genes. Expression analysis and the correlation of key DEGs will undoubtedly aid in the understanding of the roles of such genes in the development of AMI and UA. Notably, in order to get more precise correlation reports, we need to conduct a series of experimental studies to prove.

However, some limitations of our study should be considered. For instance, the differential genes analyzed by the two chips did not overlap, which may be caused by various reasons such as the source of the included samples, the annotation analysis platform, and the time of blood sampling. In addition, this study is based on the analysis of chip data; further experiments are needed to verify.

## 5. Conclusion

In summary, the integrated bioinformatical analysis could improve our understanding of DEGs between AMI and UA. The integrated bioinformatical analysis could improve our understanding of DEGs between AMI and UA.

## Figures and Tables

**Table 1:** Information of 2 microarray datasets of acute myocardial infarction and unstable angina.

Dataset	Platform	Sample (AMI/UA)
GSE29111	GPL570 [HG-U133_Plus_2] Affymetrix Human Genome U133 Plus 2.0 Array	36/16
GSE60993	GPL6884 Illumina HumanWG-6 v3.0 expression beadchip	17/9

**Table 2:** Information of key genes in PPI network of GSE29111.

Gene	UniProt ID	Degree	Expression level	Module
BDKRB2	P30411	8	Upregulation	Module 1
HRH1	P35367	6	Upregulation	Module 1
KISS1	Q15726	6	Upregulation	Module 1
GRPR	P30550	6	Downregulation	Module 1
TACR3	P29371	6	Downregulation	Module 1
HTR2B	P41595	6	Downregulation	Module 1
CCL25	O15444	3	Upregulation	—
NPBWR1	P48145	3	Upregulation	—
BDKRB1	P46663	8	Upregulation	Module 1
ORM1	P02763	2	Downregulation	Module 1

**Table 3:** Information of key genes in protein interaction network of GSE60993.

Gene	UniProt ID	Degree	Expression level	Module
IFI44	Q8TCB0	3	Downregulation	Module 1
FFAR2	O15552	2	Downregulation	—
GNG10	P50151	3	Downregulation	—
CXCR2	P25025	2	—	—
MCEMP1	Q8IX19	2	Downregulation	—
FPR2	P25090	5	—	—
CLEC4D	Q8WXI8	2	Downregulation	—
IFIT3	O14879	3	Downregulation	Module 1
RSAD2	Q8WXG1	3	Downregulation	Module 1
PLSCR1	O15162	3	Downregulation	Module 1

**Table 4:** Information on GO entries enriched by differentially expressed genes in GSE29111.

Category	ID	Item	FDR
BP	GO:0006954	Inflammatory response	6.67*E* − 04
BP	GO:0007186	G-protein-coupled receptor signaling pathway	0.004568
CC	GO:0005887	Integral component of plasma membrane	0.003999
CC	GO:0005886	Plasma membrane	0.012242

**Table 5:** Information on GO entries enriched by differentially expressed genes in GSE60993.

Category	ID	Item	*P* adjust
BP	GO:0042119	Neutrophil activation	1.41*E* − 05
BP	GO:0045088	Regulation of innate immune response	6.17*E* − 05
BP	GO:0043312	Neutrophil degranulation	6.17*E* − 05
BP	GO:0002283	Neutrophil activation involved in immune response	6.17*E* − 05
BP	GO:0002446	Neutrophil mediated immunity	6.17*E* − 05
BP	GO:0035743	CD4-positive, alpha-beta T cell cytokine production	0.000114
BP	GO:0045089	Positive regulation of innate immune response	0.000148
BP	GO:0002367	Cytokine production involved in immune response	0.000235
BP	GO:0098542	Defense response to other organisms	0.000496
BP	GO:0002369	T cell cytokine production	0.000501
BP	GO:0002720	Positive regulation of cytokine production involved in immune response	0.001009
BP	GO:0030595	Leukocyte chemotaxis	0.001785
BP	GO:0002440	Production of molecular mediator of immune response	0.001785
BP	GO:0002699	Positive regulation of immune effector process	0.001886
BP	GO:0070673	Response to interleukin-18	0.001886
BP	GO:0002526	Acute inflammatory response	0.002469
BP	GO:0002690	Positive regulation of leukocyte chemotaxis	0.002469
BP	GO:0002718	Regulation of cytokine production involved in immune response	0.002508
BP	GO:0002456	T cell mediated immunity	0.002638
BP	GO:0002708	Positive regulation of lymphocyte mediated immunity	0.002638
BP	GO:0060337	Type I interferon signaling pathway	0.002638
BP	GO:0071357	Cellular response to type I interferon	0.002638
BP	GO:0042742	Defense response to bacterium	0.002638
BP	GO:0002702	Positive regulation of production of molecular mediator of immune response	0.002638
BP	GO:0034340	Response to type I interferon	0.002648
BP	GO:0060326	Cell chemotaxis	0.002648
BP	GO:0002726	Positive regulation of T cell cytokine production	0.003238
BP	GO:0002688	Regulation of leukocyte chemotaxis	0.003238
BP	GO:0002705	Positive regulation of leukocyte mediated immunity	0.004302
BP	GO:0002687	Positive regulation of leukocyte migration	0.00459
BP	GO:0032103	Positive regulation of response to external stimulus	0.005129
BP	GO:0050921	Positive regulation of chemotaxis	0.005298
BP	GO:0009615	Response to virus	0.005298
BP	GO:0002706	Regulation of lymphocyte mediated immunity	0.005706
BP	GO:0002700	Regulation of production of molecular mediator of immune response	0.005732
BP	GO:0070102	Interleukin-6-mediated signaling pathway	0.005732
BP	GO:0002724	Regulation of T cell cytokine production	0.006379
BP	GO:0002251	Organ or tissue specific immune response	0.008387
BP	GO:0002711	Positive regulation of T cell mediated immunity	0.009107
BP	GO:0097529	Myeloid leukocyte migration	0.009288
BP	GO:0019835	Cytolysis	0.009599
CC	GO:0042581	Specific granule	1.44*E* − 05
CC	GO:0070820	Tertiary granule	1.44*E* − 05
CC	GO:0030667	Secretory granule membrane	0.000164
CC	GO:0070821	Tertiary granule membrane	0.000654
CC	GO:0035579	Specific granule membrane	0.001009
CC	GO:0098802	Plasma membrane receptor complex	0.003883
CC	GO:1904724	Tertiary granule lumen	0.009249
CC	GO:0101003	Ficolin-1-rich granule membrane	0.009249
CC	GO:0035580	Specific granule lumen	0.009249
MF	GO:0004896	Cytokine receptor activity	6.26*E* − 05
MF	GO:0019955	Cytokine binding	0.003951

## Data Availability

The raw data supporting the conclusions of this article will be made available by the authors, without undue reservation. All data obtained or analyzed during this study are available from the published article and its supplementary information files. The datasets during the current study are available from the corresponding author upon reasonable request.

## Abbreviations

5-HT: 5-Hydroxytryptamine
ACS: Acute coronary syndrome
AHA: American Heart Association
AMI: Acute myocardial infarction
BDKRB1: B1 bradykinin receptor
BDKRB2: B2 bradykinin receptor
BP: Biological process
CC: Cellular component
CCL25: C-C motif chemokine 25
CLEC4D: C-type lectin domain family 4 member D
CXCR2: C-X-C chemokine receptor type 2
DEGs: The differentially expressed genes
FC: Fold change
FFAR2: Free fatty acid receptor 2
FPR2: N-formyl peptide receptor 2
GEO: Gene Expression Omnibus
GNG10: Guanine nucleotide-binding protein G(I)/G(S)/G(O) subunit gamma-10
GO: Gene Ontology
GRPR: Gastrin-releasing peptide receptor
HRH1: Histamine H1 receptor
HTR2B: 5-Hydroxytryptamine receptor 2B
IFI44: Interferon-induced protein 44
IFIT3: Interferon-induced protein with tetratricopeptide repeats 3
IL-1*α*: Interleukin-1 alpha
IL-1*β*: Interleukin-1 beta
IL-6: Interleukin-6
IL-8: Interleukin-8
KEGG: Kyoto Encyclopedia of Genes and Genomes
KISS1: Metastasis-suppressor KiSS-1
MCC: Maximal Clique Centrality
MCEMP1: Mast cell-expressed membrane protein 1
MCODE: Molecular Complex Detection
MCP-1: Monocyte chemoattractant protein 1
MF: Molecular function
NCBI: The National Center for Biotechnology Information
NF-*κ*B: Nuclear factor-*κ*B
NIF-1: Hypoxia-inducible factor-1
NPB: Neuropeptide B
NPBWR1: Neuropeptide B/W receptor type 1
NPBWR2: Neuropeptide B/W receptor 2
NPW: Neuropeptide W
NSTEMI: Non-ST-segment elevation acute coronary syndrome
ORM1: Alpha-1-acid glycoprotein 1
PLSCR1: Phospholipid scramblase 1
PPI: Protein-protein interaction
ROS: Reactive oxygen species
RSAD2: Radical S-adenosyl methionine domain-containing protein 2
STAT3: Signal transduction and transcription activation factor 3
STEMI: ST-segment elevation acute myocardial infarction
TACR3: Neuromedin-K receptor
TCGA: The Cancer Genome Atlas
TNF-*α*: Tumor necrosis factor-*α*
UA: Unstable angina.

## Supplementary Materials

Supplementary 1 Supplementary Table 1: information of differentially expressed genes in GSE29111.

Supplementary 2 Supplementary Table 2: information of differentially expressed genes in GSE60993.
