# Supplementary material for: Biodata Mining of Differentially Expressed Genes between Acute Myocardial Infarction and Unstable Angina Based on Integrated Bioinformatics
Source: Biomed Res Int. 2021 Sep 13;2021:5584681. doi: 10.1155/2021/5584681 (PMC8456013; doi:10.1155/2021/5584681)
Supplement: Supplementary 1 — Supplementary Table 1: information of differentially expressed genes in GSE29111. [file 5584681.f1.docx]

**Supplementary Table 1.** Information of differentially expressed genes in GSE29111.

| Gene | Expression level | Gene | Expression level | Gene | Expression level |
| --- | --- | --- | --- | --- | --- |
| SLC22A11 | Up-regulation | NNMT | Up-regulation | LOC101927098 | Down-regulation |
| FABP7 | Up-regulation | LOC574538 | Up-regulation | LOC100505776 | Down-regulation |
| POLL | Up-regulation | RP11-75C9.1 | Up-regulation | RP11-513M16.7 | Down-regulation |
| CDC34 | Up-regulation | ACY1 | Up-regulation | MROH9 | Down-regulation |
| CRAT | Up-regulation | CCL25 | Up-regulation | LOC101927934 | Down-regulation |
| LOC254028 | Up-regulation | RP11-692P14.1 | Up-regulation | RP11-375I20.6 | Down-regulation |
| SMOX | Up-regulation | C3orf30 | Up-regulation | MIA2 | Down-regulation |
| TSPAN10 | Up-regulation | LINC01449 | Up-regulation | LOC100506325 | Down-regulation |
| RP11-332H18.4 | Up-regulation | STAR | Up-regulation | RP11-677O4.2 | Down-regulation |
| LINC00102 | Up-regulation | SEMG2 | Up-regulation | GPR162 | Down-regulation |
| LOC54944 | Up-regulation | PLSCR4 | Up-regulation | TBL1Y | Down-regulation |
| HRH1 | Up-regulation | ZNF683 | Up-regulation | SLC18A1 | Down-regulation |
| CD274 | Up-regulation | LINC00853 | Up-regulation | MIR663AHG | Down-regulation |
| HES6 | Up-regulation | OLFM4 | Up-regulation | LOC643549 | Down-regulation |
| ANKRD19P | Up-regulation | CATSPERD | Up-regulation | DNAJB8-AS1 | Down-regulation |
| BX648501 | Up-regulation | CABS1 | Up-regulation | ZNF474 | Down-regulation |
| NYAP2 | Up-regulation | KIRREL3 | Up-regulation | RAI2 | Down-regulation |
| RP5-991O23.1 | Up-regulation | LOC100506827 | Up-regulation | DAAM2 | Down-regulation |
| IFNK | Up-regulation | HS3ST4 | Up-regulation | HLA-DRB4 | Down-regulation |
| SFRP2 | Up-regulation | LRRC23 | Up-regulation | LOC101928101 | Down-regulation |
| NEUROG1 | Up-regulation | OR52A1 | Up-regulation | THAP7-AS1 | Down-regulation |
| RP4-742J24.2 | Up-regulation | FGF23 | Up-regulation | LOC100507377 | Down-regulation |
| TMEM54 | Up-regulation | TRIM40 | Up-regulation | SLC26A9 | Down-regulation |
| TINAGL1 | Up-regulation | LOC100287221 | Up-regulation | RP11-408I18.9 | Down-regulation |
| SMTNL1 | Up-regulation | LINC01405 | Up-regulation | LINC00410 | Down-regulation |
| C1orf87 | Up-regulation | DDX43 | Up-regulation | BTBD8 | Down-regulation |
| JAKMIP3 | Up-regulation | RP11-214N1.1 | Up-regulation | FLJ25917 | Down-regulation |
| LIN28A | Up-regulation | LOC101927746 | Up-regulation | LOC101929622 | Down-regulation |
| BC036209 | Up-regulation | RP11-303E16.7 | Up-regulation | ETNPPL | Down-regulation |
| ITLN1 | Up-regulation | DYDC1 | Up-regulation | LOC101927410 | Down-regulation |
| PAQR9 | Up-regulation | PTGES3L | Up-regulation | MYO18B | Down-regulation |
| FEV | Up-regulation | KIF24 | Up-regulation | MIR4313 | Down-regulation |
| C19orf68 | Up-regulation | LOC100129069 | Up-regulation | GPR150 | Down-regulation |
| LRRTM1 | Up-regulation | GSC2 | Up-regulation | LOC644852 | Down-regulation |
| EMX2 | Up-regulation | FETUB | Up-regulation | POU6F2 | Down-regulation |
| LOC100128993 | Up-regulation | FOXD3-AS1 | Up-regulation | MN1 | Down-regulation |
| OR1F2P | Up-regulation | SLC25A41 | Up-regulation | GRPR | Down-regulation |
| PLVAP | Up-regulation | KIAA1024 | Up-regulation | LOC285422 | Down-regulation |
| MAGEL2 | Up-regulation | MSTN | Up-regulation | SIAH3 | Down-regulation |
| FAM186A | Up-regulation | IFI27 | Up-regulation | PWAR5 | Down-regulation |
| BDKRB1 | Up-regulation | CTA-331P3.1 | Up-regulation | TDRD1 | Down-regulation |
| TEX38 | Up-regulation | RP11-619L12.4 | Up-regulation | AP000265.1 | Down-regulation |
| RP11-348B17.1 | Up-regulation | GUSBP1 | Up-regulation | TACR3 | Down-regulation |
| ABCG2 | Up-regulation | PMS2P4 | Up-regulation | DSG1 | Down-regulation |
| WFDC1 | Up-regulation | GPR15 | Up-regulation | AKR1C4 | Down-regulation |
| PRSS3P3 | Up-regulation | LOC101927667 | Up-regulation | SBSN | Down-regulation |
| MLK7-AS1 | Up-regulation | ZNF132 | Up-regulation | C2orf15 | Down-regulation |
| VMO1 | Up-regulation | LOC101927044 | Down-regulation | LOC101929765 | Down-regulation |
| NPBWR1 | Up-regulation | PNMA6A | Down-regulation | RP11-44N11.3 | Down-regulation |
| LL22NC03-N14H11.1 | Up-regulation | LPPR1 | Down-regulation | ZNF417 | Down-regulation |
| LRRC32 | Up-regulation | DIRAS3 | Down-regulation | AFM | Down-regulation |
| RP11-12M5.1 | Up-regulation | GALNTL6 | Down-regulation | LINC00632 | Down-regulation |
| KISS1 | Up-regulation | RP11-28F1.2 | Down-regulation | ARL9 | Down-regulation |
| LOC101929153 | Up-regulation | CST5 | Down-regulation | OPALIN | Down-regulation |
| LOC101929609 | Up-regulation | LINC00656 | Down-regulation | LOC101928386 | Down-regulation |
| PON3 | Up-regulation | TBX4 | Down-regulation | ZBTB12 | Down-regulation |
| TEKT3 | Up-regulation | FAM154B | Down-regulation | LOC100268168 | Down-regulation |
| ACTL8 | Up-regulation | CHI3L1 | Down-regulation | CCDC87 | Down-regulation |
| PAX8-AS1 | Up-regulation | COLEC11 | Down-regulation | MAGEA6 | Down-regulation |
| CLIP1-AS1 | Up-regulation | RP11-532F12.5 | Down-regulation | RP11-506N2.1 | Down-regulation |
| FRRS1 | Up-regulation | SCG5 | Down-regulation | LINC00567 | Down-regulation |
| LOC100129098 | Up-regulation | ORM1 | Down-regulation | IGHV3-54 | Down-regulation |
| NKX6-1 | Up-regulation | PVRL3-AS1 | Down-regulation | DEFB108B | Down-regulation |
| C15orf26 | Up-regulation | LOC101928525 | Down-regulation | KIF26A | Down-regulation |
| LOC102723684 | Up-regulation | NPM2 | Down-regulation | CYP4X1 | Down-regulation |
| FUZ | Up-regulation | PRO0471 | Down-regulation | F3 | Down-regulation |
| IL33 | Up-regulation | LOC101927824 | Down-regulation | ANP32C | Down-regulation |
| TMEM95 | Up-regulation | CYP2B7P | Down-regulation | SRGAP2 | Down-regulation |
| LOC101929027 | Up-regulation | IFNA2 | Down-regulation | RP11-50B3.4 | Down-regulation |
| CPA1 | Up-regulation | SERPINA9 | Down-regulation | ALOX15 | Down-regulation |
| RP11-343H5.6 | Up-regulation | CLDN2 | Down-regulation | HTR2B | Down-regulation |
| MUC7 | Up-regulation | ZNF674 | Down-regulation | LINC00327 | Down-regulation |
| CDC14C | Up-regulation | LINC01305 | Down-regulation | CYP4F11 | Down-regulation |
| C10orf107 | Up-regulation | EHMT1-IT1 | Down-regulation | PPP1R10 | Down-regulation |
| LL0XNC01-116E7.2 | Up-regulation | RP5-1098D14.1 | Down-regulation | RP11-138I17.1 | Down-regulation |
| EPS8L3 | Up-regulation | C19orf80 | Down-regulation | LOC285000 | Down-regulation |
| MIR622 | Up-regulation | LOC101928371 | Down-regulation | LOC101929504 | Down-regulation |
| DNM1 | Up-regulation | FOXA3 | Down-regulation | SPRR2C | Down-regulation |
| PAMR1 | Up-regulation | TVP23A | Down-regulation | RP4-633H17.2 | Down-regulation |
| LOC101927278 | Up-regulation | LDHC | Down-regulation | MAP7D2 | Down-regulation |
| BDKRB2 | Up-regulation | CTD-2293H3.1 | Down-regulation |  |  |
