# Supplementary material for: Biodata Mining of Differentially Expressed Genes between Acute Myocardial Infarction and Unstable Angina Based on Integrated Bioinformatics
Source: Biomed Res Int. 2021 Sep 13;2021:5584681. doi: 10.1155/2021/5584681 (PMC8456013; doi:10.1155/2021/5584681)
Supplement: Supplementary 2 — Supplementary Table 2: information of differentially expressed genes in GSE60993. [file 5584681.f2.docx]

**Supplementary Table 2.** Information of differentially expressed genes in GSE60993.

| Gene | | Expression level | Gene | Expression level | Gene | Expression level | |
| --- | --- | --- | --- | --- | --- | --- | --- |
| ITGB7 | Up-regulation | MCTP2 | Down-regulation | LOC651559 | Down-regulation |  |  |
| EOMES | Up-regulation | PLSCR1 | Down-regulation | IL8RB | Down-regulation |  |  |
| GZMK | Up-regulation | IL6R | Down-regulation | IFI44 | Down-regulation |  |  |
| GZMH | Up-regulation | LOC652878 | Down-regulation | ARPC1B | Down-regulation |  |  |
| MEGF9 | Down-regulation | CLEC4D | Down-regulation | FCGR3B | Down-regulation |  |  |
| HIST1H2AC | Down-regulation | LOC399744 | Down-regulation | MCEMP1 | Down-regulation |  |  |
| JUNB | Down-regulation | LOC642103 | Down-regulation | FFAR2 | Down-regulation |  |  |
| LOC653371 | Down-regulation | IRAK3 | Down-regulation | IFIT3 | Down-regulation |  |  |
| VNN2 | Down-regulation | PGAM1 | Down-regulation | FPRL1 | Down-regulation |  |  |
| LYZ | Down-regulation | LOC644774 | Down-regulation | GNG10 | Down-regulation |  |  |
| IL18R1 | Down-regulation | JAK1 | Down-regulation | LOC652616 | Down-regulation |  |  |
| PBEF1 | Down-regulation | ACSL1 | Down-regulation | IL18RAP | Down-regulation |  |  |
| LOC644063 | Down-regulation | NCF1 | Down-regulation | LOC644037 | Down-regulation |  |  |
| LOC643319 | Down-regulation | RSAD2 | Down-regulation | EIF1AY | Down-regulation |  |  |
| ECHDC3 | Down-regulation | FOLR3 | Down-regulation |  |  |  |  |
